# The management of a cystic hepatic lesion ruptured in the bile ducts: a case report

**DOI:** 10.1186/s13256-017-1329-9

**Published:** 2017-06-16

**Authors:** Hicham Baba, Mohamed Said Belhamidi, Mohammed El Fahssi, Jihad El Ghanmi, Aziz Zentar

**Affiliations:** 1 Department of General Surgery, Mohammed V Teaching Military Hospital, Faculty of Medicine and Pharmacy, Mohammed V University, Rabat, Morocco; 2 Department of Urology, University Hospital of Avicenne, Faculty of Medicine and Pharmacy, Mohammed V University, Rabat, Morocco

**Keywords:** Biliary cystadenoma, Biliary tract, Obstructive jaundice

## Abstract

**Background:**

Hepatic cystadenoma is a rare benign cystic tumor; it tends to recur after incomplete surgical resection and has malignant potential. We report the case of a patient with a ruptured biliary cystadenoma in the common bile duct that caused diagnostic and therapeutic problems.

**Case presentation:**

A 34-year-old North African woman, admitted for angiocholitis, was operated 2 months before for a hepatic cystic lesion taken for a hydatid cyst compressing her common bile duct. The clinical and the complementary examinations converged toward recurrence of the hydatid cyst for which a surgical resection was decided. Intraoperative findings as well as the histological study of the “membranes” extracted from her common bile duct indicated a hepatic cystadenoma.

**Conclusions:**

The rarity of hepatic cystadenoma and the non-specificity of clinical and imaging signs make diagnosis of hepatic cystadenoma difficult, especially when it is complicated by rupture in the bile ducts; this contributes to a delay in diagnosis and an inadequate therapeutic approach.

## Background

Hepatic cystadenoma is a rare benign cystic tumor, which most often arises from the intrahepatic biliary system; it is characterized by the risk of recurrence, in cases of incomplete resection, and malignant transformation. A lack of clinical and biological specificities makes preoperative diagnosis difficult, posing a differential diagnosis problem with other cystic lesions. Its evolution can be marked by complications; the most serious complication is a malignant transformation into cystadenocarcinoma, the risk of which is estimated to be 10 to 20% [1]. In some rare cases, the cystadenoma opens into the bile ducts and the mucinous fluid that flows into the biliary tree may cause biliary obstruction.

## Case presentation

A 34-year-old North African woman with no specific past medical history was hospitalized, initially in another hospital, for the management of obstructive jaundice with a hepatic cystic lesion of segment I taken for a hydatid cyst that compressed the biliary convergence. She had undergone a resection of the protruding dome by laparotomy. Two months later, she was admitted for angiocholitis due to a probable recurrence of the hydatid cyst complicated by compression or rupture in the bile ducts. She was febrile 38.5 °C with mucocutaneous jaundice, dark urine, and discolored stools. A clinical examination found neither defense nor hepatomegaly or palpable mass.

Biological examinations revealed moderate cytolysis and cholestasis. Hydatid serology and serum tumor markers were negative: carcinoembryonic antigen (CEA) and carbohydrate antigen (CA).

A computed tomography (CT) scan showed a hepatic cystic lesion of segment I, with parietal and septal enhancement after agent contrast injection; this lesion was ruptured in the biliary convergence.

**Fig. 1:**
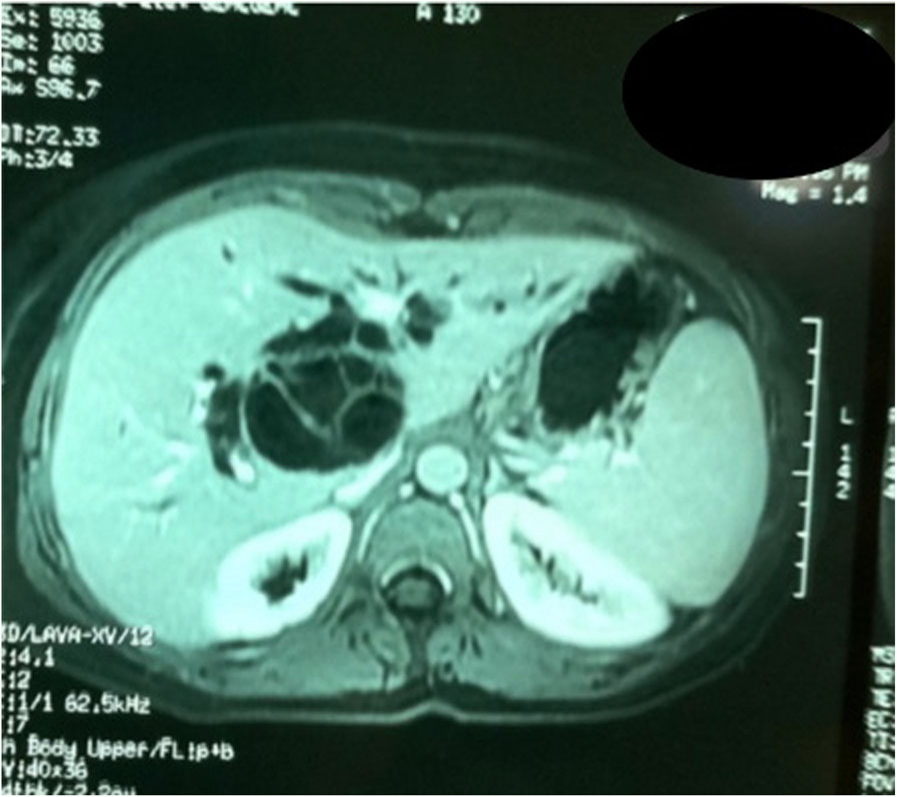
Partitioned cystic lesion of segment I of the liver with dilated intrahepatic bile ducts

**Fig. 2:**
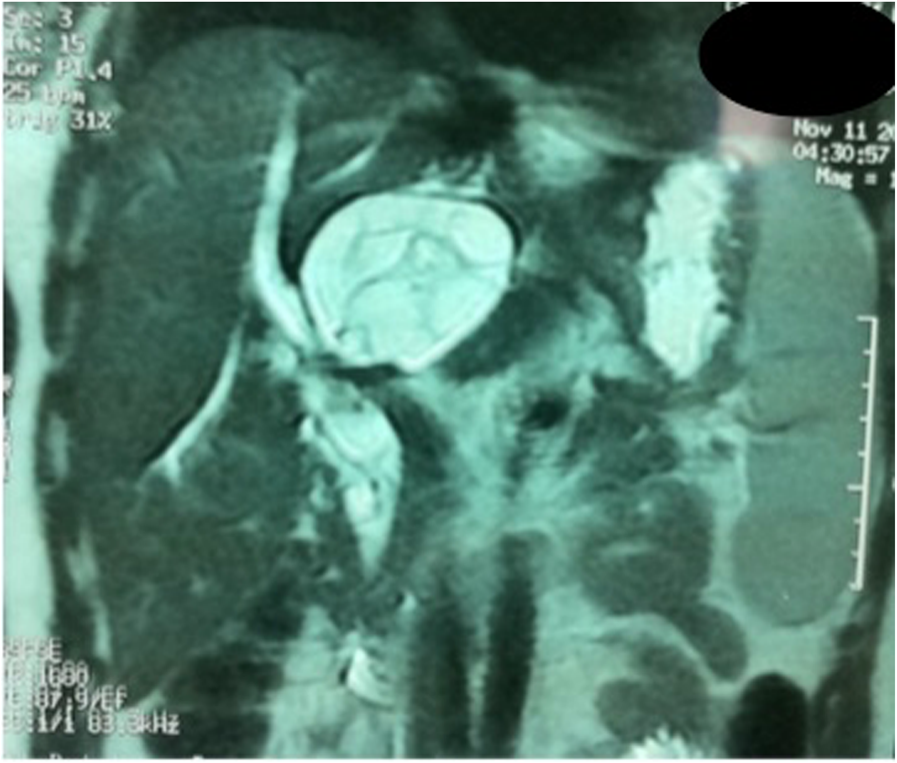
Cystic lesion ruptured in biliary convergence with extension in the common bile duct

**Fig. 3:**
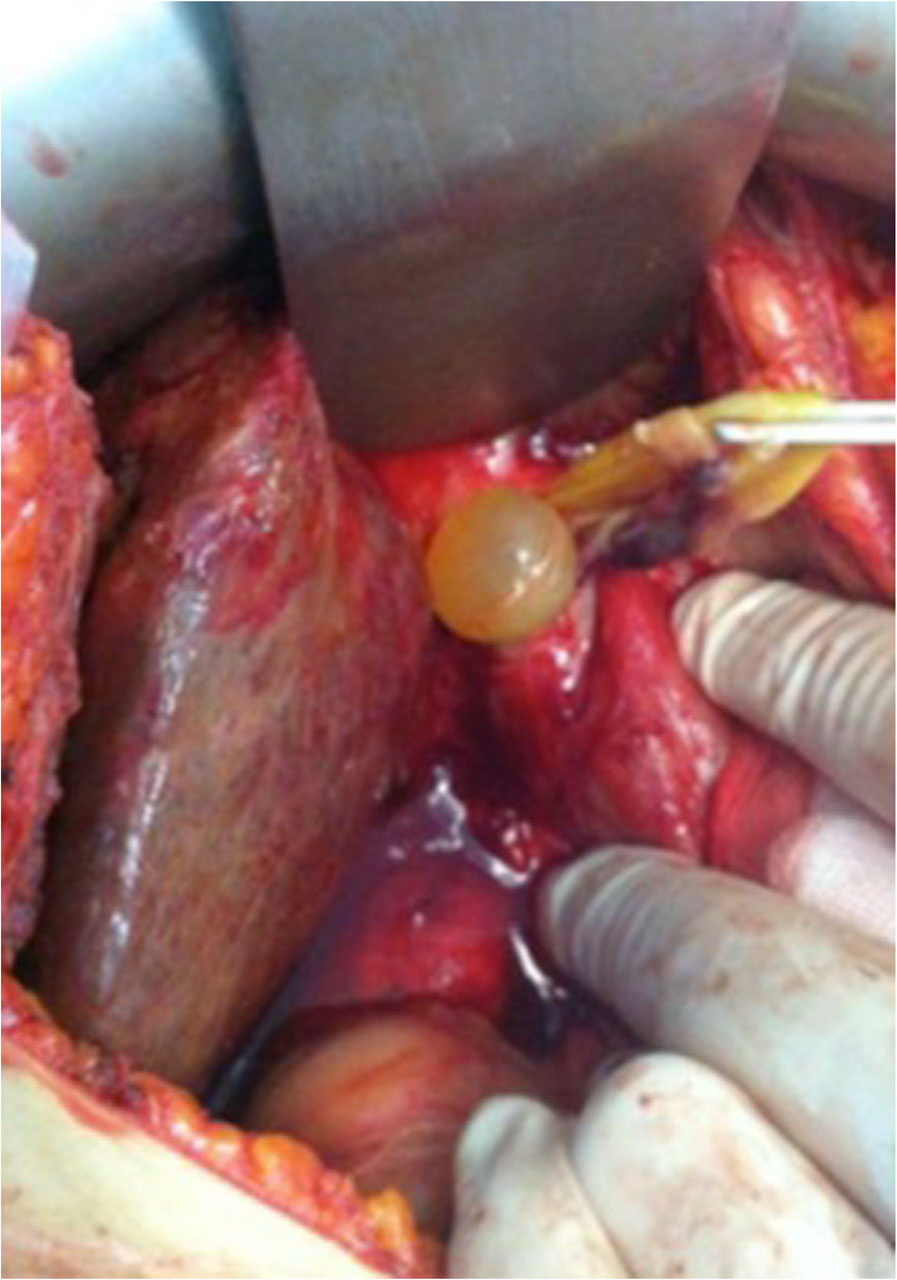
Operative view after opening of the main bile duct showing vesicles with mucinous contents as well as membranes traversed by vessels

**Fig. 4:**
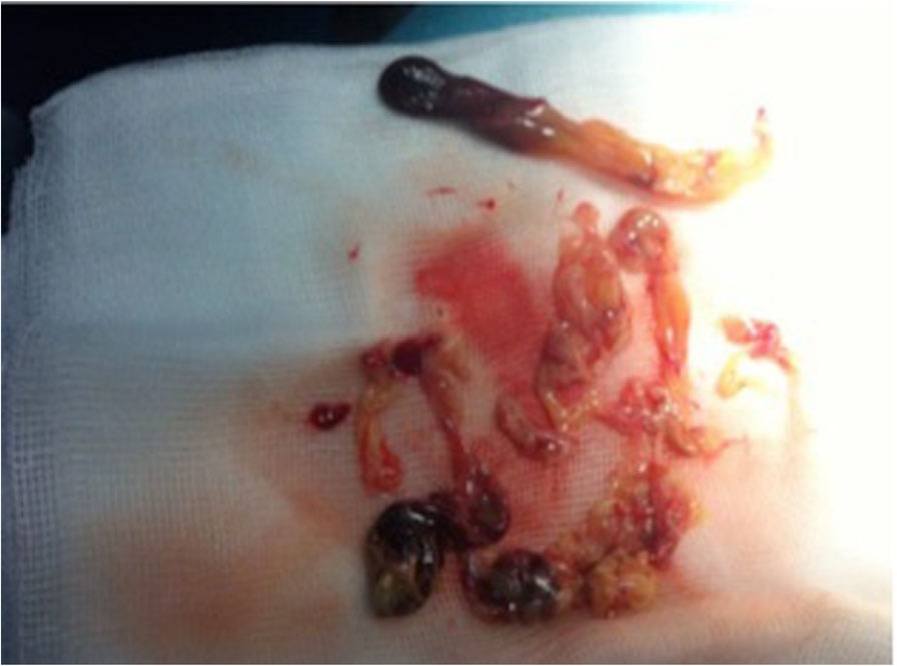
Content of the cyst (membranes)

**Fig. 5:**
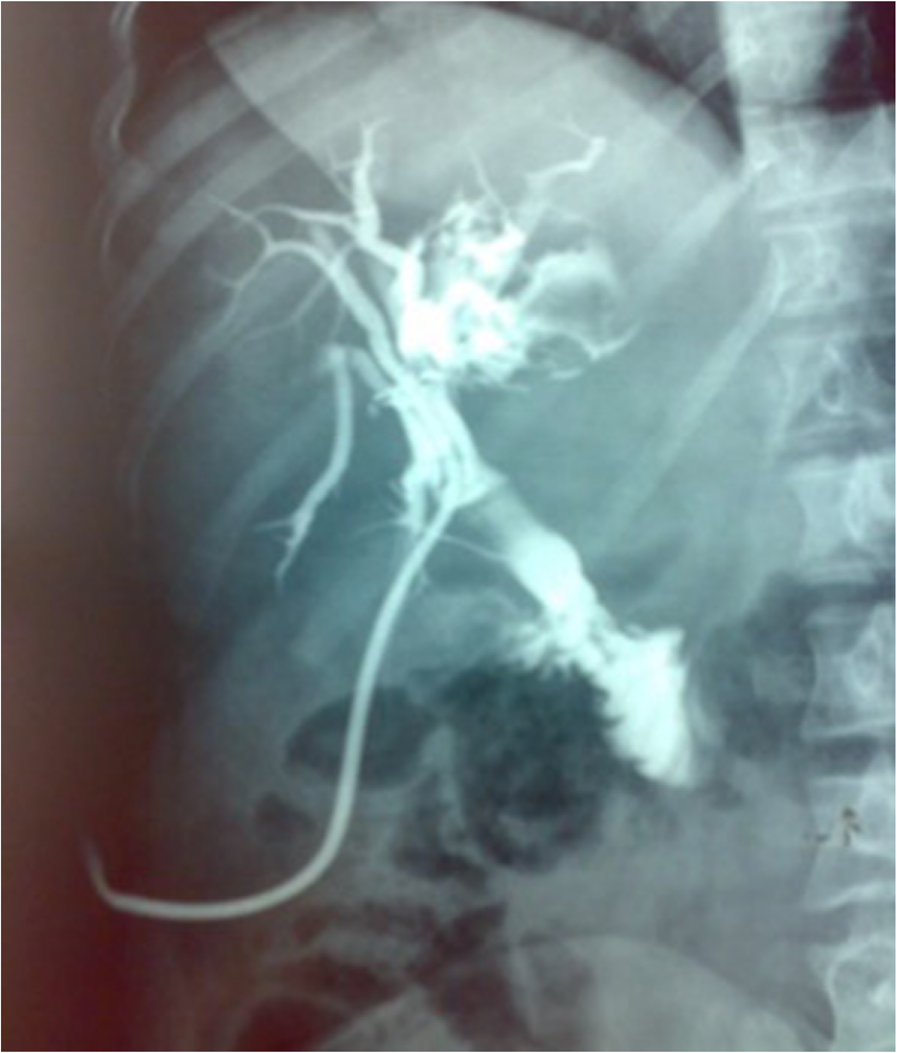
Postoperative cholangiography showing the persistence of the biliary cystadenoma

Magnetic resonance imaging (MRI) showed a partitioned lesion, hypointense in T1 and hyperintense in T2, ruptured in the biliary bifurcation and causing dilatation of intrahepatic bile ducts, especially on the left side; the common bile duct (CBD) was also dilated with heterogeneous signal content (Figs. 1 and 2). She underwent laparotomy by subcostal incision; the exploration noted a micronodular liver and the opening of her CBD allowed the extraction of membranes with a mucinous fluid issue, which raised questions about the diagnosis of hydatid cyst (Figs. 3 and 4). A t-tube was placed in the CBD at the end of the operation. A histological study of the membranes as well as liver biopsies indicated a biliary cystadenoma without signs of malignancy associated to biliary cirrhosis. Postoperative cholangiography performed 1 month later revealed the persistence of the biliary cystadenoma (Fig. 5).

Three months after this second surgery, we decided to perform radical excision after regression of jaundice and amelioration of blood tests. In fact, she underwent a left hepatectomy enlarged to segment I by laparotomy, because the cyst was ruptured in the posterior wall of the left hepatic canal, with large fistula, near the biliary convergence; a simple enucleation was impossible due to posterior location of the cyst and the adhesions of anterior surgeries. Her postoperative follow-up was simple and a histological study of the specimen confirmed the diagnosis of biliary cystadenoma without signs of malignancy.

## Discussion

Hepatic cystadenomas are extremely rare, accounting for less than 5% of non-parasitic hepatic cystic lesions [2]. The mean age of onset is between 20 and 80 years with a peak incidence for the fifth decade. Women are affected in 90 to 95% of cases [3].

Cystadenomas occur in the liver in 90% of cases; they are much less frequent in the extrahepatic bile ducts. Concurrent intrahepatic and extrahepatic cases have also been reported [4]. They are usually multilocular, with septations delimiting vesicles, and they are surrounded by a dense cellular fibrostroma. They may be unilocular, especially in the case of a small tumor [5]. They often contain a clear or mucinous fluid; hemorrhagic fluid is rare and should cause suspicion of malignancy. A bilious content is also very unusual, evoking a possible communication with the bile ducts.

They are generally asymptomatic, in the patent forms, and apart from the complications the clinical signs are not specific: hepatic colic or vague abdominal pain. In addition, a large tumor can cause a mass effect on the stomach or duodenum causing nausea, vomiting, dyspepsia, and anorexia. Abdominal palpation may reveal a moving mass with respiration.

Many complications can occur during evolution, including biliary obstruction that may be due to external biliary compression or internal obstruction by a protrusion of the cyst within the bile duct, but more rarely secondary to mucous plugs hindering bile flow; secondary biliary cirrhosis is likely to be the consequence of prolonged obstruction of the CBD.

Biology is usually normal; an increase in the activity of gamma-glutamyltransferase (GGT) and alkaline phosphatases can occur in cases of compression or obstruction of the biliary tract, leukocytosis can also be found in cases of cyst’s superinfection or angiocholitis.

In our patient, biology noted cholestasis and moderate cytolysis; the latter can be explained by the association with biliary cirrhosis.

Serum CA 19-9 analysis may be useful, but does not allow the diagnosis of cystadenoma. In fact, it can be elevated in cases of cholestasis and is therefore less reliable for the diagnosis. Analysis of CEA and CA 19-9 in the cyst fluid is more useful than serum [6]. High levels in the cystic fluid confirm the neoplastic nature and biliary origin of these cysts; their measurement has been advocated as a complementary preoperative procedure to improve the accuracy of differentiation between cystadenoma and other hepatic cystic lesions [7]. However, high intracystic levels of CEA and CA 19-9 do not prejudge the benign or malignant nature of the lesion [8].

Radiology imaging is important in diagnosis and characterization of hepatic cystic lesions. However, the radiological aspects of benign and malignant lesions overlap considerably. On ultrasound, a cystadenoma usually presents as a limited and large mass, with a thick and irregular wall that is rarely fine and smooth with internal echogenic septa, which delineate vesicles of different sizes and shapes; Wall nodules and papillary projections in the interior of the cyst can also be seen [9]. The contents of the cysts may be anechoic, hypoechoic, or echogenic depending on their nature: serous, mucinous, biliary, hemorrhagic, or mixed [10]. Bile ducts dilatation may also be present either by direct tumor extension in bile ducts or by extrinsic compression. The presence of visible mucin, as hyperechogenic material, within bile ducts could lead to a rupture of the cystadenoma in the bile ducts [11].

**Fig. 6:**
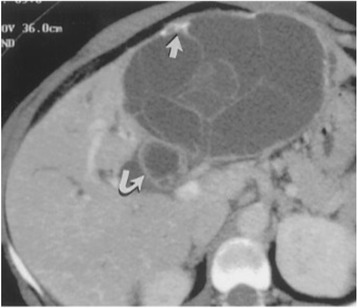
Computed tomography scan shows a multilocular cyst with septations and mural calcifications (*straight arrow*) with biliary duct dilatation and extension of the cyst into the left hepatic and common bile ducts (*curved arrow*) [9]

A CT scan determines size, morphology, and relationships with neighboring structures; sometimes it may show the extent of the cyst in the bile ducts. A CT scan shows a hypodense cystic mass, well limited, multilocular with vesicles of variable density due to a different content of mucin. The wall is thick with internal partitions and sometimes wall nodules (Fig. 6). After injection of a contrast product, an enhancement of the wall and septa as well as the possible fleshy contingent of the cyst, make the tumor more visible [11].

MRI is very specific for diagnosis and differentiation of cystadenoma from other lesions; in addition, the combination of MRI with bili-MRI is even more useful. On T1 sequences, MRI reveals a multilocular mass, partitioned with a liquid content in hyposignal. On T2 sequences, the liquid is hyperintense but heterogeneous due to a variable protein content from one vesicle to another, whereas the wall is hypointense. The intracystic septa are also hypointense in T2. After an injection of gadolinium, an enhancement of the capsule and septa, the existence of nodules, and local invasion of the liver, bile ducts, and portal vessels are suggestive of carcinomatous transformation [10].

A bili-MRI provides information on the state of the biliary tract, and the site and nature of a possible biliary obstacle: mucin, cyst’s protrusion in the bile ducts, or extrinsic compression.

In our patient, this examination made it possible to demonstrate the biliary communication and the lesion’s extension in the bile duct.

**Fig. 7:**
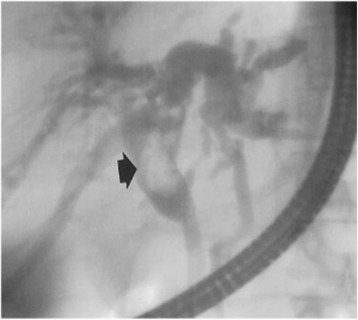
Endoscopic retrograde cholangiopancreatography image shows dilatation of the left hepatic and common ducts. There is a filling defect (*arrow*) in the common duct from intraductal extension of the cystadenoma [9]

Endoscopic retrograde cholangiopancreatography (ERCP) and percutaneous transhepatic cholangiography (PTHC) may show a heterogeneous aspect in the bile duct that may be related to the tumor or to intraluminal mucin [12] (Fig. 7); sometimes, direct communication between the tumor and the bile ducts can be seen, the endoscopic visualization of the flow of mucin through the ampulla of Vater is rare [13].

Percutaneous aspiration of the cyst with a fine needle under echographic control allows a bacteriological, cytological, and tumor markers study of the suction liquid. The cystic fluid may be hemorrhagic, bilious, mucinous, clear, or mixed; hemorrhagic fluid leads to a cystadenocarcinoma, on the other hand, a mucinous content is mostly a characteristic of biliary cystadenoma [14]. This examination can lead to the diagnosis of cystadenocarcinoma or cystadenoma, following very high levels of CEA and CA 19-9. It also enables the elimination of a hydatid cyst in its seronegative form by showing the absence of hooks and membranes [8]. In addition, it allows analysis of the bilirubin level; a high level of bilirubin raises suspicion for a communication between the cystadenoma and the bile ducts [7]. A cytological study has a sensitivity of approximately 66% for the diagnosis of malignancy but a specificity of 100%. The limits of this procedure are dissemination risk and the possibility of missing microscopic foci of malignancy in the cystadenoma [14].

The differential diagnosis of cystadenoma occurs mainly with other hepatic cystic lesions, especially with the simple cyst. Also, hepatorenal polycystic disease is a frequent hereditary disease where lesions have the same characteristics as simple cysts; the association with a polycystic kidney is constant which makes it possible to pose the diagnosis [15]. In our patient, a hydatid cyst was the main differential diagnosis due to the endemic context. The diagnostic problem becomes more difficult with type III and IV hydatid cysts, especially when the serology is negative (5 to 15% of cases) [16].

In the anatomopathologic stage, the differential diagnosis of a cystadenoma is cystadenocarcinoma. In fact, cystadenocarcinoma shares most of the morphological and radiological characteristics of cystadenoma, so the distinction is almost exclusively anatomopathologic; therefore, distinction requires search for the criteria of malignancy in multiple histologic sections [17].

The treatment of choice of hepatic cystadenomas is surgical resection. The objective is to avoid local recurrence and malignant transformation; this can be achieved by widespread surgery removing the whole cyst with a healthy tissue margin around the lesion, and making an extemporaneous examination if necessary. Enucleation may be an alternative for superficial lesions and large central lesions that are closely related to the main vascular and biliary structures.

The procedure can be performed by laparotomy or laparoscopy. The procedure is carefully selected according to the size and the location of the tumor; laparoscopic treatment may lead to results that are similar to those of open surgery [18].

Intraoperative cholangiography should be performed to search for possible biliary communication. In this case, resection of the tumor must be completed by a suture of the fistula or even a resection of the affected bile duct with a biliodigestive reconstruction if necessary.

Hepatic transplantation may be necessary in the rare cases of bilobar tumoral extension making it impossible to perform a resection [19].

In our patient, the endemic context and the radiological images led to the mistaken diagnosis of a ruptured hydatid cyst in the biliary tract; consequently, her former two operations were inadequate and it was only after histologic study of the “membranes” that the correct diagnosis was made.

The prognosis of hepatic cystadenomas is extremely good after complete resection. Inappropriate treatment, in particular aspiration or partial resection, is associated with a recurrence rate greater than 90% and a malignant degeneration risk of 30% [20].

## Conclusions

Hepatic cystadenoma is a rare tumor, which is often confused with other cystic tumors of the liver and the confusion can be increased when a hepatic cystadenoma is complicated by rupture in the bile ducts. Our observation is a concrete illustration of this confusion: a cystadenoma was twice taken for a hydatid cyst ruptured in the biliary tract, which is a much more frequent situation given our endemic context.

## Abbreviations

CA: Carbohydrate antigen
CBD: Common bile duct
CEA: Carcinoembryonic antigen
CT: Computed tomography
ERCP: Endoscopic retrograde cholangiopancreatography
GGT: Gamma-glutamyltransferase
MRI: Magnetic resonance imaging
PTHC: Percutaneous transhepatic cholangiography

## References

[1] Ishak KG, Willis GW, Cummins SD, Bullock AA. Biliary cystadenoma and cystadenocarcinoma. Report of 14 cases and review of the literature. Cancer. 1977;39:322–38. 10.1002/1097-0142(197701)39:1<322::aid-cncr2820390149>3.0.co;2-p 318915

[2] Benhamou JP. Traitement des tumeurs bénignes du foie. Gastroenterol Clin Biol. 1989;13:277–9. 2543604

[3] Wang C, Miao R, Liu H, Du X, Liu L, Lu X, *et al.* Intrahepatic biliary cystadenoma and cystadenocarcinoma: an experience of 30 cases. Dig Liver Dis. 2012;44:426–31. 10.1016/j.dld.2011.11.007 22169273

[4] Simmons TC, Miller C, Pesigan AM, Lewin KJ. Cystadenoma of the gallbladder. Am J Gastroenterol. 1989;84:1427–30. 2683743

[5] Umphrey HR, Wilcox CM, Vickers SM. Extrahepatic biliary cystadenoma localized to the common bile duct. Surgery. 2002;131:587–8. 10.1067/msy.2002.115935 12019417

[6] Dixon E, Sutherland FR, Mitchell P, McKinnon G, Nayak V. Cystadenomas of the liver: a spectrum of disease. Can J Surg. 2001;44:371–6. PMC3692645 11603751

[7] Koffron A, Rao S, Ferrario M, Abecassis M. Intrahepatic biliary cystadenoma: role of cyst fluid analysis and surgical management in the laparoscopic era. Surgery. 2004;136:926–36. 10.1016/j.surg.2004.06.031 15467680

[8] Bonnet S, Béchade D, Palazzo L, Desramé J, Baton O, Bounaim A, *et al.* Ponction sous échoendoscopie d’un cystadénome hépatique. Gastroentérologie Clin Biol. 2005;29:607–9. 10.1016/s0399-8320(05)82138-4 15980760

[9] Levy AD, Murakata LA, Abbott RM, Rohrmann Jr CA. From the Archives of the AFIP: Benign Tumors and Tumorlike Lesions of the Gallbladder and Extrahepatic Bile Ducts: Radiologic-Pathologic Correlation 1. Radiographics. 2002;22:387–413. 10.1148/radiographics.22.2.g02mr08387 11896229

[10] Bassou D, Darbi A, Goasdoué P, El Kharras A. Cystadénome biliaire intra-hépatique: apport de l’imagerie. Feuill Radiol. 2007;47:387–91.

[11] Manouras A, Markogiannakis H, Lagoudianakis E, Katergiannakis V. Biliary cystadenoma with mesenchymal stroma: Report of a case and review of the literature. World J Gastroenterol. 2006;12:6062-9. 10.3748/wjg.v12.i37.6062 PMC4124420 17009411

[12] Lind DS, Adolph V, Parker GA. Mucinous biliary cystadenoma: a case report and review of the literature. J Surg Oncol. 1992;5:207–10. 10.1002/jso.2930510316 1434648

[13] Hanazaki K. Intrahepatic biliary cystadenoma demonstrated by intraoperative cholangiography. Hepatogastroenterology. 1995;43:1024–8. 8884333

[14] Kim K, Choi J, Park Y, Lee W, Kim B. Biliary cystadenoma of the liver. J Hepatobiliary Pancreat Surg. 1998;5:348–52. 10.1007/s005340050058 9880787

[15] Vilgrain V. Lésions kystiques du foie. Gastroenterol Clin Biol. 2001;25:167–77. 11449158

[16] Cadranel JF, Benhamou JP. Cystadénome du foie. Hépato-Gastro Oncol Dig. 1998;5:109–14.

[17] Owono P, Scoazec JY, Valette PJ, Dumortier J, Gouysse G, Berger F, *et al.* Cystadénomes et cystadénocarcinomes hépatobiliaires: Etude clinique, radiologique et anatomopathologique de 7 cas. Gastroentérologie Clin Biol. 2001;25:414–21. 11449129

[18] Shaked O, Siegelman ES, Olthoff K, Reddy KR. Biologic and clinical features of benign solid and cystic lesions of the liver. Clin Gastroenterol Hepatol. 2011;9:547–62. 10.1016/j.cgh.2011.03.007 21397723

[19] Romagnoli R, Patrono D, Paraluppi G, David E, Tandoi F, Strignano P, *et al.* Liver transplantation for symptomatic centrohepatic biliary cystadenoma. Clin Res Hepatol Gastroenterol. 2011;35:408–13. 10.1016/j.clinre.2011.03.014 21549659

[20] Morris MW, Anderson CD, Drake LC, Redfield SM, Subramony C, Vanderlan WB. Giant biliary cystadenoma. J Surg Case Rep. 2012;2012(9):15. 10.1093/jscr/2012.9.15 PMC3649621 24960801

